# A Comparison of Change Blindness in Real-World and On-Screen Viewing of Museum Artefacts

**DOI:** 10.3389/fpsyg.2018.00151

**Published:** 2018-02-16

**Authors:** Jonathan E. Attwood, Christopher Kennard, Jim Harris, Glyn Humphreys, Chrystalina A. Antoniades

**Affiliations:** ^1^NeuroMetrology Group, Nuffield Department of Clinical Neurosciences, University of Oxford, Oxford, United Kingdom; ^2^Medical Sciences Division, University of Oxford, Oxford, United Kingdom; ^3^Ashmolean Museum Engagement Programme, Ashmolean Museum of Art and Archaeology, University of Oxford, Oxford, United Kingdom; ^4^Department of Experimental Psychology, University of Oxford, Oxford, United Kingdom

**Keywords:** change blindness, vision, perception, real-world, on-screen, binocular, museum, artefact

## Abstract

Change blindness is a phenomenon of visual perception that occurs when a stimulus undergoes a change without this being noticed by its observer. To date, the effect has been produced by changing images displayed on screen as well as changing people and objects in an individual’s environment. In this experiment, we combine these two approaches to directly compare the levels of change blindness produced in real-world vs. on-screen viewing of museum artefacts. In the real-world viewing condition, one group of participants viewed a series of pairs of similar but slightly different artefacts across eye saccades, while in the on-screen viewing condition, a second group of participants viewed the same artefacts across camera pans on video captured from a head-mounted camera worn by the first set of participants. We present three main findings. First, that change blindness does occur in a museum setting when similar ancient artefacts are viewed briefly one after another in both real-world and on-screen viewing conditions. We discuss this finding in relation to the notion that visual perceptual performance may be enhanced within museums. Second, we found that there was no statistically significant difference between the mean levels of change blindness produced in real-world and on-screen viewing conditions (real-world 42.62%, on-screen 47.35%, *X*^2^ = 1.626, *p* > 0.05 1 d.f.). We discuss possible implications of these results for understanding change blindness, such as the role of binocular vs. monocular vision and that of head and eye movements, as well as reflecting on the evolution of change detection systems, and the impact of the experimental design itself on our results. Third, we combined the data from both viewing conditions to identify groups of artefacts that were independently associated with high and low levels of change blindness, and show that change detection rates were influenced mainly by bottom-up factors, including the visible area and contrast of changes. Finally, we discuss the limitations of this experiment and look to future directions for research into museum perception, change blindness, real-world and on-screen comparisons, and the role of bottom-up and top-down factors in the perception of change.

## Introduction

Change blindness is defined as the failure to detect when a change is made to a visual stimulus (Simons and Levin, 1997). It occurs when the local visual transient produced by a change is obscured by a larger visual transient, such as an eye blink (O’Regan et al., 2000), saccadic eye movement (Grimes, 1996; McConkie and Currie, 1996), screen flicker (Rensink et al., 1997), or a cut or pan in a motion picture (Simons, 1996; Levin and Simons, 1997); or when the local visual transient produced by a change coincides with multiple local transients at other locations, known as mud-splashes, which act as distractions, causing the change to be disregarded (O’Regan et al., 1999). Change blindness is distinct from inattentional blindness, which occurs when an individual is blind to the presence of an entire object while performing a distracting task [as in the well-known “gorilla in the room” experiment (Simons and Chabris, 1999)]. In contrast, change blindness occurs when an individual is blind to changes occurring to an object with which they are actively engaged. Because of this, when missed changes are later pointed out to the observer, they are usually met with a sense of disbelief at how something could ever have been missed. The surprising nature of change blindness results from a disconnect between the assumption that our visual perceptions are so detailed as to be virtually complete, and the actual ability of the visual system to represent and compare scenes moment-to-moment. In this way, change blindness is a testable phenomenon that can be used to investigate the nature of visual representations in different conditions (Simons and Rensink, 2005).

In most of the studies published to date, change blindness has been produced using altered photographs or videos of natural scenes displayed on computer screens. More recently, change blindness has also been shown to take place in more naturalistic scenarios. For example, in one real-world experiment, more than half of participants failed to notice the changing of a conversation partner in front of them (Simons and Levin, 1998; Levin et al., 2002), and in another, more than half of participants were blind to the changing of an object’s colour or a printed word’s font (Varakin et al., 2007).

In the current experiment, we sought first to demonstrate whether change blindness could be produced inside a museum, using ancient museum artefacts as visual stimuli. It has been suggested that the visual interactions taking placed within museums involve enhanced perception compared to everyday visual interactions (O’Neill and Dufresne-Tassé, 1997), raising the question of whether change blindness is still a demonstrable phenomenon under such conditions. Inattentional blindness has been previously investigated in a museum setting (Levy, 2011), but as far as we are aware this is the first attempt to produce change blindness inside a museum.

Once it has been produced, we will directly compare the levels of change blindness produced by a single set of visual stimuli viewed in both on-screen and real-world conditions. In the real-world condition, one group of participants viewed a series of pairs of similar but slightly different artefacts across eye saccades, while in the on-screen condition, a second group of participants viewed the same series of artefacts across camera pans on video captured from a head-mounted camera worn by the first set of participants. It is important to know whether or not this shift to more on-screen interaction has negative consequences such as increased change blindness. To the best of our knowledge, this is the first attempt to directly compare change blindness levels produced in on-screen and real-world viewing conditions.

Our motivation for making this comparison was twofold. First, as a response to the relative lack of comparisons between on-screen and real-world perception made to date, despite the extensive use of both conditions across human visual perception research. Because non-stereoscopic cameras capture and display light from a single perspective, on-screen viewing conditions provide only monocular cues to visual depth. These depth cues include linear perspective, object occlusion, and motion parallax (Cutting, 1997; Albertazzi et al., 2010). By contrast, because in real-world viewing conditions light reflected from the three-dimensional environment is captured from the perspective of both eyes without passing through a camera, binocular depth cues, including binocular disparity and ocular convergence, become available in addition to the monocular cues. There is evidence to suggest that binocular stereoscopic vision confers an advantage over monocular vision in certain perception performance tasks, including the analysis of complex visual scenes (Jones and Lee, 1981), surface visualisation (Wickens et al., 1994), and the programming of prehensile movements (Servos et al., 1992). However, evidence of preserved function without stereopsis also exists, most notably amongst pilots (Snyder and Lezotte, 1993), and the overall functional significance of binocular stereopsis remains unclear (Fielder and Moseley, 1996). Based on this evidence and our own observations, our hypothesis is that change blindness levels will be lower in the real-world condition than in the on-screen condition, because the perceptual advantages of binocular over monocular vision will produce a greater rate of change detection in the real-world scenario.

We were also motivated to make this comparison by the increasing frequency and importance of on-screen visual interactions alongside real-world interactions in modern working and social life. The growing accessibility of high-speed internet and the capability of smart portable devices has already significantly changed the way that many people exchange visual information. A recent report found that adults in the United States spend an average of more than 8 h a day accessing media through a device with a screen (The Nielsen Total Audience Report - Q1 2016, 2016). For many people, this amount of time will account for the majority of their waking day and such a significant shift in behaviour warrants further investigation in its own right.

## Materials and Methods

We recruited 62 participants through an advertisement describing a neuropsychological experiment taking place at the Ashmolean Museum in Oxford. The group of participants consisted of students and employees of the University of Oxford, covering a wide range of disciplines from Art History and Fine Art to Law and Medicine. While none of the participants were artists, they might all be considered to hold some form of interest in art, or art history, given that they responded to our advert. The participants were allocated using a random number generator to either real-world or on-screen viewing conditions. 31 participants were allocated to each group. The mean age of participants in the real-world group was 22.8 years (SD ± 5.3 years) and 58.1% were female. The mean age of participants in the on-screen group was also 22.8 years (SD ± 5.9 years) and 58.1% were female. No attempt was made to match the groups. The exact sex matching occurred by chance. The close age matching results from the participants predominantly being university students. This study was carried out with permission from the Central University Research Ethics Committee (CUREC), and all subjects gave their written informed consent after the experimental procedures had been explained to them, in accordance with the Declaration of Helsinki.

### Museum Setting

The experiment was conducted in the Ashmolean Museum of Art and Archaeology, part of the University of Oxford. Twelve pairs of artefacts from the museum’s collection were used, including three pairs of Japanese woodblock prints, one pair of Chinese porcelain bowls, two pairs of Iranian tiles, one pair of Athenian lekythoi, one pair of Renaissance bronze medals, two pairs of Anglo-Saxon brooches, and two pairs of English silverware. These artefacts were chosen because although they had originally been designed to appear identical in their pairs, through their individual manufacture and subsequent usage they had all come to exhibit differences, ranging from relatively subtle to more major differences in appearance, including differences in colour, shape, and design. There were differences between all 12 pairs of artefacts used in the experiment.

### Change Blindness Paradigm

Twelve pairs of artefacts were displayed in a fixed order before each participant. For each pair of artefacts, a participant observed one item for a short period of time before looking to the second item and observing it for the same length of time as the first. As participants looked from one item to the next, the differences between their appearances generated local visual transients. However, the transition of looking from one item to the other generated a larger visual transient which would to a certain extent obscure the local transients, and thus produce a corresponding degree of change blindness. This degree was measured by participants responding to the question: *Did you notice any differences between the two objects?* They were then required to describe any differences they did notice in writing after viewing each pair of artefacts. Subsequently, the participants’ descriptions were marked as either correct or incorrect according to the actual differences manifest between the objects. If none of the changes existing between a pair of artefacts were correctly identified, the participant was recorded as being change blind with respect to that pair. If a single change was correctly identified, they were recorded as not being change blind. The degree of change blindness recorded was therefore a reflection of the balance of local and large visual transients that were produced by observing these pairs of museum artefacts in real-world and on-screen viewing conditions.

The length of time for which participants observed each artefact was set at a duration that would produce a change blindness effect appropriate to allow for a comparison to be made between the two conditions. The requisite duration was determined through a series of trials in which photographs of the pairs of artefacts were observed in series on a monitor for different lengths of time. An observation time of 5 s per artefact separated by an interval of 2 s resulted in change blindness in 15% of the pairs. Observation time of 2 s with an interval of 0.5 s produced change blindness in 20%, and an observation time of 0.25 s with an interval of 0.25 s produced 57% change blindness. Given that the motion of turning to look from one artefact to another would produce an interval between fixations of less than 100 ms (Grossman et al., 1988), an observation time of 1 s was chosen in order to achieve approximately 50% mean change blindness in the on-screen condition. This was thought to be optimal in allowing for a comparison to be made between this and the real-world condition.

**FIGURE 1:**
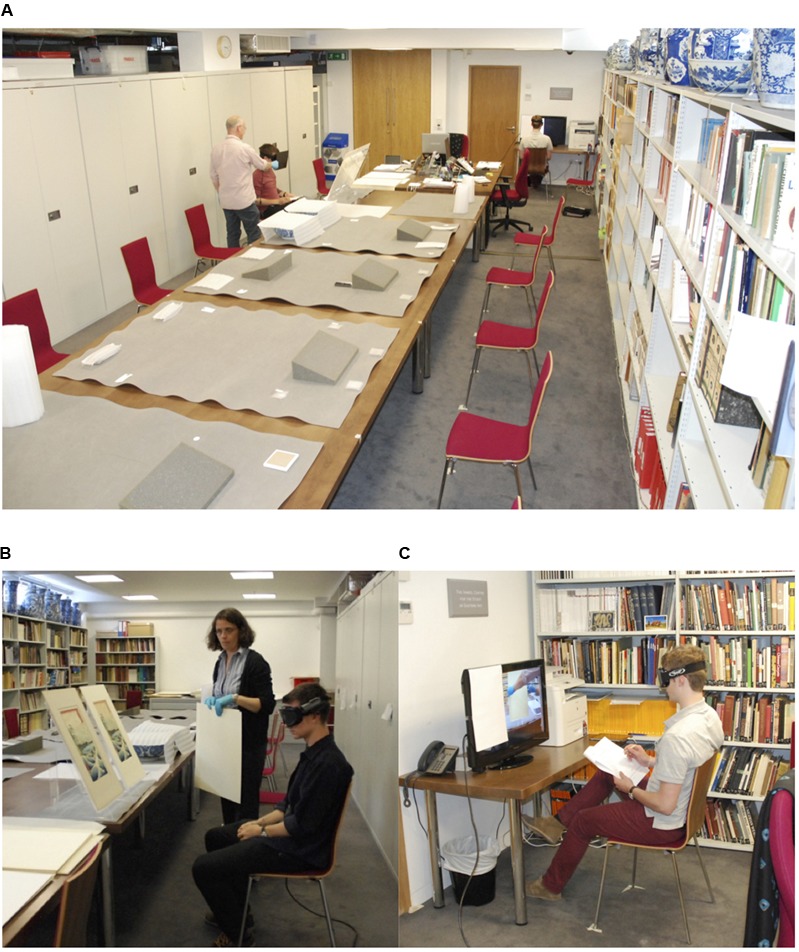
**(A)** The experimental setup within the museum, showing the artefacts (covered), two participants, and an experimenter. **(B)** The real-world viewing condition: the participant is sat in front of a pair of artefacts, wearing a pair of modified goggles and head-mounted camera. **(C)** The on-screen viewing condition, the participant is sat in front of a monitor, wearing a pair of modified goggles and watching a live feed from the head-mounted camera. Images reproduced with permission from Ashmolean Museum, University of Oxford. All the persons depicted on this picture gave their consent for publication.

Both viewing conditions were similarly controlled to standardise the nature and duration of the periods of observation, and the transition from one artefact to another. The artefacts were placed in their pairs on a table in a room within the museum (**Figure 1A**). They remained covered for the majority of the experiment, and members of museum staff were present to ensure their safekeeping throughout. The items in each pair were placed 40 cm apart, and a chair was placed in front of each pair of artefacts to provide a viewing distance of 75 cm. A high definition 32-inch LCD screen was also present in the room with a chair placed in front of it. The real-world viewing condition consisted of participants sitting in front of and viewing the artefacts on the table before them (**Figure 1B**). The on-screen condition consisted of a separate group of participants sitting in front of the screen and viewing the artefacts on its display (**Figure 1C**). Both participants were aware of each other and their roles throughout the course of the experiment.

**FIGURE 2:**
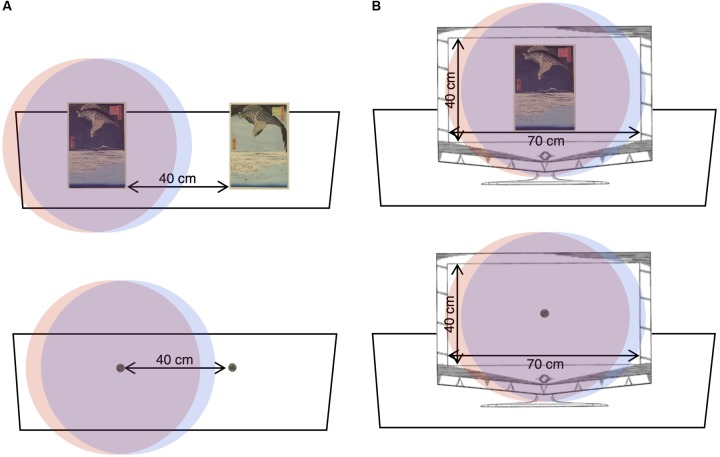
**(A)** The real-world participants’ views of the largest (top) and smallest (bottom) artefacts through the modified goggles. The whole surface of the largest artefact was visible, but both items of the smallest pair of artefacts were not visible at the same time (to scale). **(B)** The on-screen participants’ views of the largest (top) and smallest (bottom) artefacts through the modified goggles on the screen (to scale). Images reproduced with permission from Ashmolean Museum, University of Oxford.

All participants’ visual fields were restricted by wearing a pair of goggles that were modified for the purposes of this study. Opaque inserts were fitted to the inside of the goggles to leave a window of 3 cm diameter in front of each eye. This restricted the binocular field of view to 45.56° (0.79°rad) horizontally and 48.14° (0.84 rad) vertically at the 75 cm viewing distance. The field was sufficient to contain the full surface of the largest artefact while also not allowing both of the smallest artefacts to be viewed when the visual field was centred on one of them, in both the real-world (**Figure 2A**) and on-screen conditions (**Figure 2B**). These steps were taken to ensure that participants would not be able to make multiple eye saccades between the items in front of them, which would have added a significant uncontrolled variable.

### Real-World Viewing Condition

Once sat in front of the first pair of artefacts, the real-world participant was instructed to start with their head toward the item on their left, so that their visual field would be centred on the first artefact. The artefact was initially obscured by a small screen. On an auditory cue the screen was manually removed by an experimenter so that the participant could view the first artefact. This period of observation lasted for 1 s, after which another cue sound signalled for the participant to turn their head and eyes to look at the second item to their right, so that their visual field would now be centred on the second artefact. This period of observation lasted for a further 1 s, after which a small screen was placed between the participant and the second item by an experimenter so that it could no longer be seen. In this way, both artefacts were viewed for a duration of 1 s, with a brief visual transition interrupting the viewings.

The visual transition which occurred in the real-world viewing condition consisted of a combination of a head rotation and a saccadic eye movement. This combination has been defined elsewhere as a gaze shift (Binder et al., 2009), where gaze is defined as the sum of eye position with respect to the head and head position with respect to the body. When the visual field shifts more than 15–20°, an eye saccade is normally accompanied by a head rotation in order to return the eyes to a neutral position within the orbits and allow the extra-ocular muscles to relax. In this case, the shift was 28.1° (0.49 rad), and participants in the real-world condition were specifically instructed to turn both their head and eyes to view the second artefact in each pair.

The coordination of gaze shifts is complex but the basic elements are well-understood (Pelisson and Guillaume, 2009). As the head initially rotates and the eyes stay fixed on the first target, eye movement is under the control of the vestibulo-ocular reflex (VOR). Once head rotation has brought the new target into the visual field, an endogenous eye saccade occurs to move the point of foveation from the first target to the second. Following this, though the second target is now foveated, there is still residual head rotation due to a lag in the control of head movement relative to that of the eyes, and this is compensated for by a further period of VOR eye movement. The components of the gaze shift are therefore an initial period of VOR, an exogenous eye saccade, followed by a further period of VOR. It is not yet known whether VOR eye movements are able to induce change blindness by themselves, but that eye saccades are able to is well-established (Grimes, 1996; McConkie and Currie, 1996). Thus, in the real-world viewing condition in this experiment, the large visual transient consisted of an eye saccade which was preceded and followed by a period of VOR eye movement.

### On-Screen Viewing Condition

While the above processes were taking place, a small head-mounted high definition video camera was attached to the goggle strap of the participant in the real-world viewing condition. The camera used was a Contour+2 HD with 170° wide-angle lens, operating at a frame rate of 30 fps and 1920 × 1080 resolution, weighing 156 g, and measuring 98 mm × 60 mm × 34 mm. It was connected by an HDMI cable to 1080p high definition 32-inch LCD screen, producing a live video feed on the screen in front of the participant in the on-screen condition. The acuity achievable when viewing this screen was 20/70, which, although inferior to 20/20 vision, was significantly greater than the level required to resolve the smallest change detected by any participant in the real-world condition, which was measured to be 20/180 (a change of 2 mm diameter viewed at 75 cm). The on-screen participant wore an identical pair of modified goggles to their counterpart in the real-world group (except without a camera attached to the goggle strap), which, as in the real-world group, prevented multiple eye saccades being made between artefacts.

Unlike participants in the real-world viewing condition, however, on-screen participants did not have to follow instructions to move their head or eyes on auditory cues. Instead, as the real-world participant rotated their head to look from the first item to the second, the head-mounted camera also rotated and the footage on the screen panned across to reveal the second artefact to the on-screen participant. An equivalent change to the contents of the visual field was therefore produced without an equivalent gaze shift taking place. Thus, in the on-screen viewing condition, the large visual transient consisted of a camera pan rather than an eye saccade preceded and followed by a period of VOR. Of course, the other difference between viewing conditions was that artefacts were viewed directly by participants in the real-world group, while they were viewed on an LCD display in the on-screen group. On-screen participants viewed the screen from a distance of 75 cm, and the camera and screen were calibrated so that the representations of the artefacts were displayed at life-size in order to match conditions in the real-world conditions. In both real-world and on-screen viewing conditions, therefore, the artefacts subtended the same visual angle.

The only differences between the conditions, then, were the nature of the large visual transient and the format of display. We suggest that these variables constitute the defining differences between all real-world and on-screen visual interactions, in that they represent both the behaviour of the subject who is viewing and the nature of the object that is being viewed in these scenarios. Thus, the results of this experiment reflect a comparison of the levels of change blindness produced by a single set of visual stimuli in real-world and on-screen viewing, as defined by the nature of the large visual transient and the format of display typical of these conditions.

## Results

**FIGURE 3:**
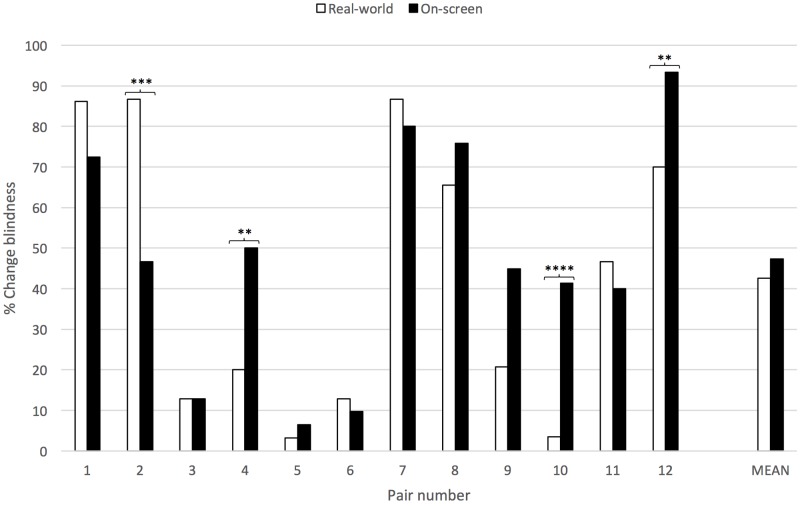
Levels of change blindness in real-world and on-screen viewing conditions produced by each pair of artefacts and the overall mean. Asterisks denote level of significance (*X*^2^ test with one degree of freedom. No asterisk = *p* > 0.05; ^∗^ = 0.05 > *p* > 0.02; ^∗∗^ = 0.02 > *p* > 0.01; ^∗∗∗^ = 0.01 > *p* > 0.001; ^∗∗∗∗^ = 0.001 > *p*).

We present three main findings. First, that change blindness does occur in a museum setting when similar ancient artefacts are viewed briefly one after another in both real-world and on-screen viewing conditions (**Table 1** and **Figure 3**).

**Table 1:** Table of results.

Pair no.	Total trials	Real-world			On-screen			Real-world v. On-screen		Combined RW and OS
		Change detected	Change blind	Change blind (%)	Change detected	Change blind	Change blind (%)	*X*^2^	*P*-value	Mean change blind (%)
1	29	4	25	86.2	8	21	72.4	1.681	0.194	79.31
2	30	4	26	86.7	16	14	46.7	10.800	0.001	66.67
3	31	27	4	12.9	27	4	12.9	0.000	1.000	12.90
4	30	24	6	20.0	15	15	50.0	5.934	0.015	35.00
5	31	30	1	3.2	29	2	6.5	0.350	0.554	4.84
6	31	27	4	12.9	28	3	9.7	0.161	0.688	11.29
7	30	4	26	86.7	6	24	80.0	0.480	0.488	83.33
8	29	10	19	65.5	7	22	75.9	0.749	0.387	70.69
9	29	23	6	20.7	16	13	44.8	3.835	0.050	32.76
10	29	28	1	3.5	17	12	41.4	11.997	0.001	22.41
11	30	16	14	46.7	18	12	40.0	0.271	0.603	43.33
12	30	9	21	70.0	2	28	93.3	5.455	0.020	81.67
Total	359	206	153		189	170				
**Mean**				**42.62**			**47.35**	**1.626**	**0.202**	**44.97**

*RW, real-world; OS, on-screen. X^2^ test with one degree of freedom.*

Second, we found that there was no statistically significant difference between the mean levels of change blindness produced in real-world and on-screen viewing conditions [real-world 42.62%, on-screen 47.35%, *X*^2^= 1.626, *p* > 0.05 1 d.f. (**Table 1** and **Figure 3**)]. The total number of trials per pair of artefacts ranged from 29 to 31 due to a small number of failures by participants to follow the experimental procedure described above (13 failures from 371 trials = 3.5%). The mean level of change blindness produced in the on-screen condition was close to 50%, as intended to allow comparison between the two conditions. One pair of artefacts produced a significantly higher degree of real-world change blindness than on-screen change blindness (Pair 2: real-world 86.7%, on-screen 46.7%, *X*^2^ = 10.800, 0.01 > *p* > 0.001), while three pairs produced a significantly higher degree of on-screen change blindness than real-world change blindness (Pair 4: real-world 20.0%, on-screen 50.0%, *X*^2^= 5.934, 0.02 > *p* > 0.01; Pair 10: real-world 3.5%, on-screen 41.4%, *X*^2^ = 11.997, 0.001 > *p*; Pair 12: real-world 70.0%, on-screen 93.3%, *X*^2^= 5.455, 0.02 > *p* > 0.01). But in the other eight pairs, and overall, there was no significant difference between the levels of change blindness produced.

**FIGURE 4:**
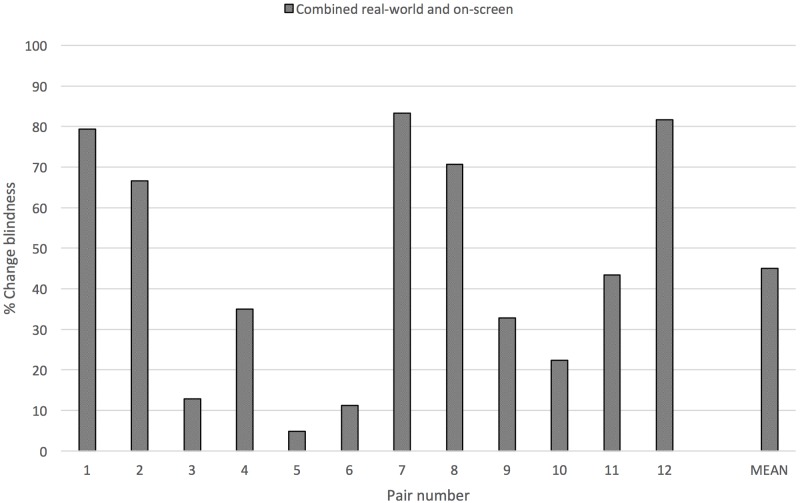
Levels of change blindness combined from real-world and on-screen viewing conditions produced by each pair of artefacts and the overall mean.

Third, following the finding of no significant difference between the levels of change blindness produced in real-world and on-screen conditions, we combined the data from both groups to compare the levels of change blindness produced by each pair of artefacts independently (**Figure 4**). From these results, we consider in particular three pairs of artefacts which produced a level of change blindness greater than 75% (pairs 1, 7, and 12, 79.31–83.33%), and three pairs of artefacts which produced a level of change blindness lower than 15% (pairs 3, 5, and 6, 4.84–12.90%).

## Discussion

### Change Blindness in a Museum Setting

Our first finding, that change blindness does occur in a museum setting when similar ancient artefacts, in this case some more than 2,000 years old, are viewed briefly one after another in both real-world and on-screen viewing conditions, is a significant addition to the body of evidence demonstrating that change blindness can be produced in more naturalistic environments outside of the laboratory.

Change blindness experiments have established that details considered to be important are detected more readily than those that are less important (Rensink et al., 1997; O’Regan et al., 2000), even when the changes are of equivalent physical salience (Kelley et al., 2003). These findings suggest that attention plays an important role in prioritising the elements of a visual scene, and in determining what is represented and compared between scenes and what is not (Simons and Rensink, 2005). However, even changes that are made to attended objects can still be missed (Ballard et al., 1995; Simons, 1996), which leads to the conclusion that attention is necessary, but not sufficient, for change detection to occur. The other determinants of change detection can be divided between bottom-up, or stimulus-driven, factors, such as visual salience, and top-down, or goal-driven, factors, such as context, gist and motivation (Borji and Itti, 2013). It has been suggested that both bottom-up and top-down factors are enhanced in the visual interactions that take place within museums, due to the exceptional and exemplary nature of the objects being viewed, and the intensity of observation and motivation to form interpretations from what is seen, respectively (O’Neill and Dufresne-Tassé, 1997). However, later discussions have warned against ‘uncritical acceptance of the distinction between utilitarian (ordinary) and the aesthetic (museum) seeing,’ and while it is acknowledged that ‘the notion of the distinction…is, in one form or another, firmly embedded in many account of vision and aesthetic experience,’ in fact, ‘cognitive neuroscience does not supply any facts that could substantiate the sharp divide between the ‘normal’ and the aesthetic perception’ (Kesner, 2006). In the present experiment, we did not compare the levels of change blindness produced within and outside of the museum setting. Instead, we have merely demonstrated that change blindness can be produced among participants viewing artefacts inside a museum. However, in light of this finding and the unresolved questions that surround it, such a comparison would be an appropriate next step.

### Change Blindness in Real-World and On-Screen Viewing Conditions

Our second finding was that there was no statistically significant difference between the mean levels of change blindness produced in real-world and on-screen viewing conditions. This means that altering both the format of display from the objects themselves to an on-screen virtual object representation, and the nature of the large visual transient from a camera pan to an eye saccade preceded and followed by VOR, did not significantly affect the rate of change detection in this experiment.

The possible interpretations of this finding are: (1) that neither the format of display nor the nature of the large visual transient had a significant effect on change detection, (2) that the format of display and nature of the large visual transient had equal and opposite effects on change detection, resulting in no combined effect overall, or (3) that the similarities between the two conditions were so great compared to the differences, that any effects produced by either the format of display or the nature of the large visual transient were masked by the intrinsic design of the experiment.

Regarding the first part of the first interpretation, if the format of display had no significant effect on change detection, then this finding provides no support for our hypothesis, which was that the perceptual advantages of binocular stereoscopic vision would produce a greater rate of change detection in the real-world condition compared to the on-screen condition.

Our hypothesis was formulated based on evidence that binocular stereoscopic vision confers an advantage over monocular vision in certain perception performance tasks, including the analysis of complex visual scenes (Jones and Lee, 1981), surface visualisation (Wickens et al., 1994), and the programming of prehensile movements (Servos et al., 1992). We also made reference to the fact that in the real-world condition, binocular stereopsis would provide additional depth cues of binocular disparity and ocular convergence, compared to in the on-screen condition where only monocular depth cues of linear perspective, object occlusion, and motion parallax would be available (Cutting, 1997; Albertazzi et al., 2010). According to our first interpretation, this finding runs contrary to the evidence supporting our initial hypothesis. However, due to the equal plausibility of the other interpretations, we cannot reliably contrast our finding with those drawn elsewhere. As previously discussed, evidence of preserved visual performance without stereopsis does exist (Snyder and Lezotte, 1993), and so the overall functional significance of binocular stereopsis must unfortunately remain unclear.

In the absence of clear evidence either way, it is interesting and perhaps instructive to consider the evolutionary arguments for why we might expect change detection to be enhanced by binocular stereoscopic vision. One could argue that a real-world object, with the potential to act upon its viewer and itself to be acted upon, should be perceived more strongly than an on-screen object, which ultimately remains virtual (although the screen which displays it is itself a real-world object). However, as some of the artefacts used in this experiment demonstrate, the human visual system has been processing two-dimensional representations of three-dimensional objects for thousands of years. The earliest cave paintings discovered date back to 15,000–10,000 BC. Human beings, and especially the human nervous system, have undergone significant changes over hundreds of generations in this time, but we reason that in this period there will have been no drive to either significantly strengthen or weaken the local visual transients formed from the observation of two-dimensional images relative to three-dimensional objects. The subjects of the earliest two-dimensional representations were bison, mammoth, and reindeer, the prey of those who depicted them on the walls of their dwellings. This alone is testament to the fact that the ability to create and understand representations of the surrounding environment and the messages being communicated about them is likely to have conferred a selective advantage over the recent course of our evolution. Indeed, while objects that can act upon us and that we can act upon have remained important for our survival, one can argue that images have come to be just as important to the modern human.

Returning to the second part of the first interpretation of our finding, it is possible that the nature of the large visual transient had no significant effect on change detection. We are not aware of any previous attempts to compare the effect of eye saccades and VOR vs. camera pans on visual performance. And, as above, due to the equal plausibility of the other interpretations, we cannot present this interpretation as a reliable conclusion. Theoretically, one could reasonably argue that activation of neural systems controlling head and eye movements might either enhance or impair the parallel systems involved in change detection. Once again, in the absence of evidence, we might have recourse to consider evolutionary arguments. However, in this situation this seems hardly relevant. Before motion pictures were developed at the turn of the twentieth century, the human visual system would never have been exposed to a change to the contents of the visual field in the absence of head or eye movements – such a thing would simply not have been possible. Consequently, there has been almost no time for natural selection to affect the mechanisms of change detection operating in the context of a camera pan compared to an eye saccade accompanied by VOR eye movement.

The second possible interpretation of our finding is that the format of display and nature of the large visual transient had equal and opposite effects on change detection, resulting in no combined effect overall. Because of the difficulties in drawing conclusions about either variable discussed above, the uncertainty of following this interpretation would be even greater, and as such need not be discussed further.

The third possible interpretation of our main finding was that the similarities between the two conditions were so great compared to the differences, that any effects produced by either the format of display or the nature of the large visual transient were masked by the intrinsic design of the experiment. In any experiment, the pattern of findings will be determined by a balance between both the controls and the variables that constitute the experimental paradigm (Gozli, 2017). Our paradigm included a relatively large number of controls and restrictions: a fixed viewing time, a restricted field of view, proscribed head and eye movements, and a specific set of visual stimuli. It was necessary to institute these limitations to reliably isolate our two experimental variables from a complex naturalistic scenario. However, it is possible that, such was the impact of these controls relative to the difference between the experimental variables, that our two viewing conditions were in effect much more similar than they were different. In this way, it is possible that the similarity in task performance across the two conditions could have masked effects produced by the differences between on-screen and real-world viewing conditions. It is perhaps not possible to determine to what degree any effects may have been masked. However, with this in mind, we can only state that the differences between on-screen and real-world viewing conditions were not large enough to produce a significant difference in participant performance in the context of this experiment.

In summary, then, it is difficult to interpret our finding that there was no statistically significant difference between the mean levels of change blindness produced in real-world and on-screen viewing conditions. The effects of altering the format of display and the nature of the visual transient in this experiment cannot be separated, the possibility of equal and opposite effects cannot be excluded, and the possibility that effects were masked by the overall similarity of the viewing conditions must be considered.

### Bottom-Up and Top-Down Factors

The third and final finding of this study came after combining the data across both conditions to compare the levels of change blindness produced by each pair of artefacts independently. We consider in particular three pairs of artefacts which produced a level of change blindness greater than 75% (pairs 1, 7, and 12, 79.31–83.33%), and three pairs of artefacts which produced a level of change blindness lower than 15% (pairs 3, 5, and 6, 4.84–12.90%). Given that the nature of the large visual transients was controlled across the experiment, it follows that these data reflect the fact that local visual transients produced by the changes between the artefacts in pairs 1, 7, and 12 were weaker than those produced by the changes between the artefacts in pairs 3, 5, and 6. These local transients arose from the differences in appearance of the pairs of artefacts.

**FIGURE 5:**
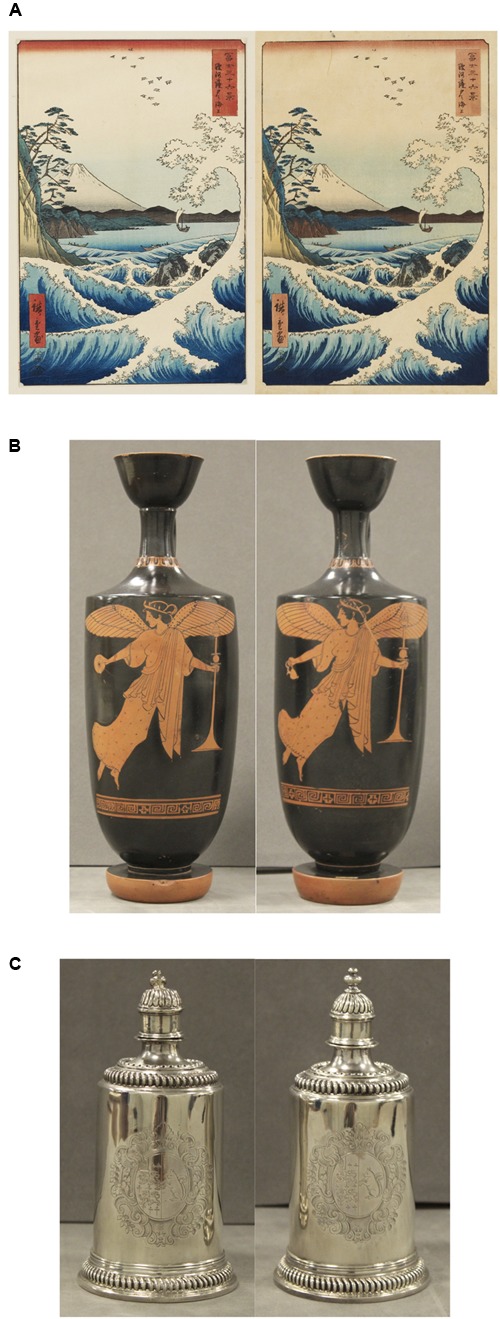
**(A)** Pair 1: Utagawa Hiroshige, *The Sea at Satta in Suruga Province* from *Thirty Six Views of Mount Fuji*. Woodblock prints with *bokashi* (tonal gradation). 1858-9 AD. 22.4 cm × 34.0 cm. **(B)** Pair 7: Athenian red-figure lekythoi. *Nike flying with phiale* (left). *Nike flying with thurible* (right). 490–480 BC. 32.4 cm (left) and 31.8 cm (right) tall. Images reproduced with permission from Ashmolean Museum, University of Oxford. **(C)** Pair 12: Isaac Dighton, Silver toilet dressing table service, 2 of 14. 1699–1700 AD. 10.3 cm (left) and 10.5 cm (right) tall. Images reproduced with permission from Ashmolean Museum, University of Oxford.

**FIGURE 6:**
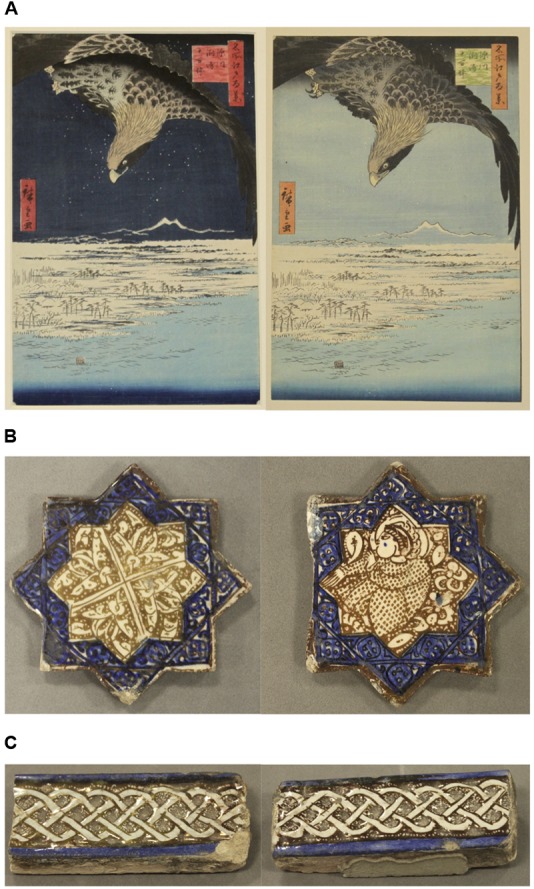
**(A)** Pair 3: Utagawa Hiroshige, *Jûmantsubo Plain at Susaki, near Fukagawa* from *One Hundred Famous Views of Edo*. Woodblock prints with *bokashi* (tonal gradation). 1856-8 AD. 22.0 cm × 32.8 cm. Images Ashmolean Museum, University of Oxford. **(B)** Pair 5: Iranian star tiles. Late 13th–14th century AD. 16.0 cm × 6.5 cm, 15.0 cm × 15.0 cm (left), 13.0 cm × 15.0 cm (right). **(C)** Pair 6: Iranian tiles with interlacing pattern. 13th century AD. Images reproduced with permission from Ashmolean Museum, University of Oxford.

Taking pairs one and three, both Japanese woodblocks prints, as an example, the artefacts in both pairs are the same size as each other and share the same designs (**Figures 5A**, **6A**). Pair one, the wave prints, also share very similar colouring (**Figure 5A**). The only differences in colouring between this pair are the subtle changes in hue to the border and box containing script. These changes in colour are slight and cover a small proportion of the visible surface of the artefacts. By contrast, pair three, the eagle prints, are more obviously different in colour (**Figure 6A**). For instance, the colour of the sky changes from dark blue to light blue between the two prints, and the colour of the boxes containing script changes from pink and red to green and orange. Collectively, these changes represent a more significant colour change and cover a larger proportion of the artefact’s visible surface, compared to the wave prints. It is these local visual transients which account for the lower level of change blindness amongst participants viewing pair three compared to pair one (12.90% vs. 79.31%, respectively).

Pair seven, the Athenian lekythoi, produced a high level of change blindness similar to that produced by pair one, the wave prints. The artefacts in this pair are the same size as each other, share the same red-figure colouring, and have near-identical designs, except for the depiction of the object in the figure’s right hand, which changed from a phiale to a thurible (**Figure 5B**). As for pair one, this change covers a small area of the visible surface of the artefacts, and so represents a relatively small local visual transient, which translates to a high level of change blindness (83.33%). Similarly, pair 12, the silver flasks, also produced a high level of change blindness (81.67%). The two flasks are practically identical, save only for the uppermost tip which has been displaced atop the first item (**Figure 5C**). Again, this change represents a small area of the artefact’s visible surface, and so only produced a small local visual transient.

The artefacts in pair six, the Iranian tiles with interlacing pattern, are the same size as each other and share the same design and colouring (**Figure 6C**). However, the first tile has an area of damage to its corner and the second tile carries an extra piece of cement on its front. These changes together account for a large area of the artefacts’ visible surfaces, and as such constitute large local visual transients responsible for a low level of change blindness (11.29%). Pair five, the Iranian star tiles, produced the lowest level of change blindness of all (4.84%), with only three of the 62 participants not noticing any changes between them. These artefacts manifest differences in both the design of their central area, and also in that one of the points on the second tile has been broken off (**Figure 6B**). These two changes constitute large local visual transients accounting for a very low level of change blindness.

The characteristics of changes that produced the most easily detected local visual transients include a large visible area of change and high contrast changes in colour. Both of these characteristics, area and contrast, can be directly related to the retina, where light from the visual field is transduced by photoreceptor cells, and contrast is enhanced by lateral inhibition of neurons in the layers between the photoreceptors and retinal ganglion cells. Because these characteristics are amongst the first to be encoded by the visual system, they are possible candidates for bottom-up influences on the prioritisation of what is represented and compared during the process of conscious change perception. In line with this, it has been shown that highly salient objects, where salience includes colour, intensity, and orientation (Koch and Ullman, 1985; Itti and Koch, 2000), attract visual fixations earlier than less salient objects (Underwood and Foulsham, 2006; Underwood et al., 2006), and it is well-established that the larger a surface is within the visual field the more likely it is to be fixated (Peschel and Orquin, 2013).

However, it is clear that areas undergoing change can be fixated within a change blindness paradigm without the change itself being perceived (O’Regan et al., 2000). It has also been shown that bottom-up factors can at times be overridden by top-down cognitive influences, such as the consistency of an object within the gist of a scene (Underwood and Foulsham, 2006; Stirk and Underwood, 2007), and the specific task the viewer is asked to perform when observing a stimulus (Underwood et al., 2006). In this experiment, there are likely to have been many top-down influences derived from the artefacts themselves, such as the prior knowledge that ancient pottery is more likely to exhibit differences in terms of damage, while prints may be more likely to exhibit colour differences. However, the two groups of artefacts which produced the lowest and highest levels of change blindness, respectively, both exhibited differences of colour, design, and damage. This suggests that top-down influences concerning types of changes had a minimal effect on the level of change blindness produced by each artefacts. For this reason, we suggest that bottom-up factors were relatively spared from being over-ridden by top-down effects, and were therefore able to exert their own influence on the processes of representation and comparison, and ultimately change blindness. In this way, our findings support a role for bottom-up factors including a large visible area of change and high contrast colour change in determining which elements in a visual scene are represented and compared in the process of conscious change perception, in both real-world and on-screen viewing conditions.

### Limitations

The methods used in this study carry their own limitations. We will discuss them in relation to the two main comparisons performed in this experiment. Namely, the comparison of real-world and on-screen viewing conditions, and the comparison of the 12 pairs of artefacts. Regarding the former, first, by comparing the performance of two different groups of participants in real-world and on-screen conditions, we introduced the potential for selection bias. We saw no practicable alternative to this, as a change cannot be shown to the same participant more than once in a change blindness experiment. To mitigate this bias, we recruited over 30 participants that we randomly allocated to each group, which resulted in near-identical demographics being represented in both.

Second, while it was important to control the conditions in which the artefacts were observed, this was at the expense of the naturalism of the viewing experience. The viewing distance and placements of the objects were similar to what would be found in a natural museum environment, but the brief periods of observation and the removal of peripheral vision using modified goggles were both unnatural. However, the conditions were the same for participants in both groups. Third, by recording changes which participants described incorrectly in the same way as changes that were not described at all, we set a relatively high threshold for change detection to be achieved. Our methodology did not distinguish between the experience of completely missing a change and the experience of sensing that a change had occurred but not being able to describe that change correctly. It is also possible that the head movement of the real-world observer provides an extra attentional cue to the on-screen observer by centering on the change.

Regarding the comparison between the 12 pairs of artefacts, first, it is possible that the performance of participants changed over the course of the experiment as they advanced through the 12 sets of observations. It is both conceivable that their performance may have improved due to a learning effect, or conversely have worsened due to fatigue. We expect that because each observation was only brief (less than 3 s), and the number of observations was relatively few, neither of these effects are likely to have impacted significantly on the levels of change blindness recorded over the course of the experiment. Each set of 12 trials took less than 10 min to perform. Although the order in which the artefacts were viewed was not varied between participants (which could have mitigated any such effects), the levels of change blindness produced from pair one to pair 12 bear no relation to either an increasing or decreasing trend. Finally, the collection of artefacts used as visual stimuli did not contain a control pair, in that there was no pair of artefacts that were truly identical to each other. If such a pair had produced a change blindness level of 100% it would have strengthened the confidence with which we can draw conclusions from our data.

## Conclusion

Change blindness is a testable phenomenon of visual perception that can be used to investigate the nature of visual perception in different conditions. It has been produced in naturalistic scenarios outside of the laboratory before using everyday objects, but until now it has not been produced in a setting such a museum, where visual perception may be enhanced. We have for the first time demonstrated that change blindness can be produced inside a museum, using ancient museum artefacts as visual stimuli, under both real-world and on-screen viewing conditions. We anticipate further experiments will be required to fully investigate the notion of altered visual perception inside museums.

While in society, on-screen interactions are increasingly coming to replace real-world ones, there is a relative lack of experimental comparisons between visual perceptual performance in real-world and on-screen conditions. We have for the first time directly compared the levels of change blindness produced by a single set of visual stimuli viewed in both on-screen and real-world conditions, and found that there was no statistically significant difference between the levels of change blindness produced in the two conditions. This does not appear to support our original hypothesis that change detection would be enhanced in real-world conditions relative to on-screen due to the perceptual advantages of binocular stereoscopic vision. We discuss the difficulty of interpreting this finding and caution against generalising the result of this experiment too readily.

In light of this finding, we combined the data from both viewing conditions to identify groups of artefacts that were independently associated with high and low levels of change blindness, and found that change detection rates were influenced mainly by bottom-up factors, including the visible area and contrast of changes, more than top-down factors. In this way, our findings support a role for bottom-up factors in determining which elements in a visual scene are represented and compared in the process of conscious change perception, in both real-world and on-screen viewing conditions. Finally we discuss the intrinsic limitations of this experiment which must be considered alongside its results. We hope, nevertheless, that our attempt to add to the understanding of visual perception within museums, the phenomenon of change blindness, perceptual performance in real-world and on-screen conditions, and the role bottom-up and top-down factors in change detection will motivate further research into these increasingly relevant questions.

## Author Contributions

JA, CK, JH, GH, and CAA designed the research, revised and improved the manuscript. JA and CAA analysed the data and prepared the figures. JA, CK, JH, and CAA discussed the results, advised on interpretation and contributed to the final draft of the manuscript. All authors contributed to and had approved the final manuscript.

## Conflict of Interest Statement

The authors declare that the research was conducted in the absence of any commercial or financial relationships that could be construed as a potential conflict of interest.
